# A redox-sensitive phosphatase regulates glycolysis as a metabolic switch in the bacterial inner membrane

**DOI:** 10.1126/sciadv.aea8724

**Published:** 2026-05-06

**Authors:** Lei Zheng, Wei Niu, Xianfa Xie, Trung Vu, Guangwei Du

**Affiliations:** ^1^ Department of Biochemistry and Molecular Biology, Center for Membrane Biology, The University of Texas Health Science Center at Houston McGovern Medical School, Houston, TX, USA.; ^2^ Department of Biology, Center for Biotechnology Genomics and Bioinformatics, Virginia State University, Petersburg, VA, USA.; ^3^ Department of Integrative Biology and Pharmacology, The University of Texas Health Science Center at Houston McGovern Medical School, Houston, TX, USA.

## Abstract

Microorganisms rapidly adjust their metabolism to survive fluctuating environmental conditions, but how they coordinate glycolytic control with redox signals remains unclear. We found that the membrane phosphatase PgpA acts as a redox-sensitive switch to regulate glycolytic flux in *Escherichia coli*. PgpA dephosphorylates key glycolytic intermediates, glyceraldehyde-3-phosphate and glycerol-3-phosphate, to modulate central metabolism. This activity is controlled by a reversible disulfide bond that forms an inactive dimer under oxidative stress and restores activity when reduced. This redox-dependent regulation enables *E. coli* to fine-tune metabolism in response to changes in nutrients and oxygen availability. PgpA inactivation increases glucose uptake and promotes metabolism, while constitutive activation impairs growth under anaerobic conditions. We also found that PgpA influences redox homeostasis by regulating glutathione biosynthesis. These findings reveal a negative feedback mechanism in which PgpA integrates glycolysis with redox balance, serving as a central regulator of bacterial metabolic homeostasis in response to environmental changes.

## Teaser

Redox-sensitive phosphatase PgpA couples glycolysis and redox balance to control metabolic adaptation in *E. coli*.

## INTRODUCTION

Bacterial survival and growth depend on their ability to adapt to fluctuating environmental conditions, including changes in nutrient availability, oxygen levels, and metabolic demands (*1*–*3*). Central to this adaptability is the precise regulation of metabolism, which ensures a dynamic balance of energy production and biosynthesis. Glycolysis, as a core metabolic pathway, plays a dual role in this balance: It generates adenosine 5′-triphosphate (ATP) and reduced form of NAD^+^ [nicotinamide adenine dinucleotide (oxidized form)] (NADH) to meet energy requirements while providing essential intermediates for biosynthetic pathways (*4*, *5*). Alterations in glycolysis can significantly influence bacterial metabolic flux, enabling cells to respond rapidly to environmental challenges (*6*, *7*).

Such metabolic adaptability is crucial for bacteria to persist and grow in hostile environments, including host immune responses and antibiotic treatments (*8*, *9*). For instance, facultative anaerobes such as *Escherichia coli* and *Pseudomonas aeruginosa* shift to anaerobic metabolism in low-oxygen conditions, thereby facilitating survival in deep tissues or biofilms (*10*). Metabolic reprogramming also plays a key role in bacterial persistence, a state often observed in chronic infections, where reduced metabolic activity renders bacteria less susceptible to antibiotics targeting actively dividing cells (*11*, *12*). Comprehending the mechanisms of metabolic regulation is essential for understanding bacterial physiology and may lead to the development of strategies for combating infections and addressing antibiotic resistance (*13*, *14*).

Redox balance is critical for regulating bacterial metabolism (*15*–*17*). Increasing evidence suggests that the intracellular redox state directly modulates glycolytic activity (*18*). For example, glycolysis is tightly coupled to the NADH/NAD^+^ ratio: When NAD^+^ is limited or NADH accumulates, glycolytic flux is attenuated (*19*). In addition, key glycolytic enzymes such as glyceraldehyde-3-phosphate dehydrogenase (GAPDH) are found to be redox sensitive in some bacteria, undergoing oxidative inactivation through cysteine modification (*20*). At the transcriptional level, global redox regulators like OxyR and SoxRS modulate the expression of metabolic genes in response to oxidative stress (*21*, *22*). Despite these insights, the mechanisms by which redox signals directly control glycolytic flux remain poorly understood.

**Fig. 1.**
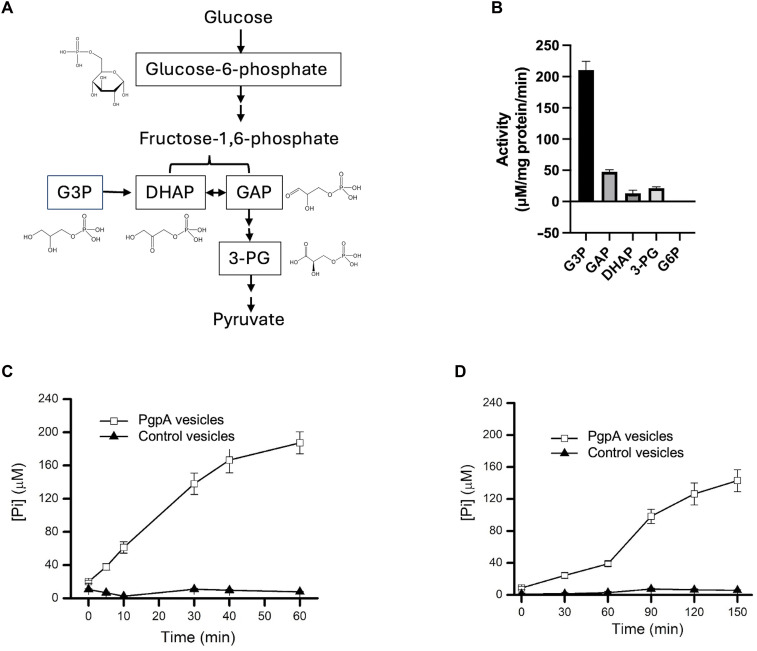
PgpA hydrolyzes GAP and G3P at the *E. coli* inner membrane. (**A**) Schematic representation of key three-carbon phosphate intermediates in glycolysis. Glucose is converted to glucose-6-phosphate (G6P) and then to fructose-1,6-bisphosphate, which is cleaved into dihydroxyacetone phosphate (DHAP) and glyceraldehyde-3-phosphate (GAP). GAP is further processed to 3-phosphoglycerate (3-PG) and then pyruvate. G3P, derived from glycerol metabolism, is converted to DHAP to enter glycolysis. Tested substrates were highlighted with their chemical structures. (**B**) In vitro phosphate release assays reveal substrate specificity of PgpA. Reactions (100 μl) containing 1 μg of purified PgpA proteins and 5 μg of each substrate were incubated at room temperature for 20 min. Activities were normalized to background levels in the individual no-enzyme control. (**C** and **D**) Time course of PgpA-dependent inorganic phosphate release ([Pi]) measured using inside-out vesicles prepared from *E. coli* BL21(DE3) cells expressing PgpA or carrying an empty vector. Vesicles were incubated with 1 mM G3P (C) or 5 mM GAP (D). Data are presented as means ± SD (*n* = 3).

Phosphatidylglycerol phosphatase A (PgpA) is an integral membrane enzyme protein. PgpA is traditionally recognized for its role in phospholipid biosynthesis, catalyzing the dephosphorylation of phosphatidylglycerol phosphate (PGP) to produce phosphatidylglycerol (PG), a primary component of bacterial membranes (*23*, *24*). We recently found that, besides its lipid phosphatase activity, PgpA hydrolyzes two glycolytic three-carbon sugar phosphate metabolites: glyceraldehyde-3-phosphate (GAP) and glycerol-3-phosphate (G3P) (Fig. 1A) (*25*). These two metabolites can be used not only for energy production but also as precursors for the synthesis of macromolecules and lipids (*4*, *5*, *26*). This finding suggests that PgpA functions as a bridge between lipid biosynthesis and central carbon metabolism. However, its physiological role in regulating glycolysis and broader metabolic homeostasis remains unknown.

In this study, we identify a role for PgpA as a metabolic regulator that integrates glycolytic flux and redox homeostasis in *E. coli*. Using biochemical assays, mutational analysis, and metabolomic profiling, we demonstrate that PgpA dynamically regulates glycolytic flux to balance biosynthesis and energy production using a disulfide switch on the cytoplasmic membrane surface. This mechanism enables *E. coli* to fine-tune its metabolism in response to environmental changes, such as nutrient availability and oxygen levels. This redox regulatory mechanism is likely conserved across Gram-negative microorganisms, highlighting its evolutionary significance in maintaining bacterial metabolic homeostasis.

## RESULTS

### PgpA hydrolyzes GAP and G3P on the cytoplasmic membrane surface

PgpA is one of three PG phosphatases in *E. coli*, along with PgpB and PgpC (*23*). However, maintaining any one of these three enzymes is sufficient to sustain PG levels comparable to wild type, indicating functional redundancy in PG biosynthesis (*23*). Although all three enzymes catalyze the dephosphorylation of PGP, we recently reported that PgpA uniquely hydrolyzes the soluble PGP headgroup analogs GAP and G3P (*25*). This finding suggested that PgpA may function as a moonlighting enzyme involved in both bacterial lipid and glycolysis metabolism. To confirm and extend this observation, we examined PgpA toward a range of glycolytic phosphate intermediates, including three-carbon and six-carbon phosphate metabolites (Fig. 1A). In vitro phosphate release assays were consistent with our previous findings and revealed robust activity toward GAP and G3P, whereas activity toward dihydroxyacetone phosphate (DHAP) and 3-phosphoglycerate (3-PG) was significantly weaker, and no activity was detected toward glucose-6-phosphate (Fig. 1B). These results confirm the substrate specificity of PgpA for three-carbon sugar phosphate metabolites GAP/G3P.

To validate this activity in a native membrane environment, we measured GAP and G3P phosphatase activity using crude membrane vesicles generated from *E. coli* BL21(DE3) cells expressing PgpA. As the catalytic pocket of PgpA is predicted to face the cytoplasmic side of the membrane, vesicles were prepared in an inside-out orientation using a low-pressure method to ensure uniform topology. Vesicles expressing PgpA efficiently hydrolyzed GAP and G3P, whereas no activity was detected in control vesicles lacking PgpA (Fig. 1, C and D). These results confirm that PgpA dephosphorylates these glycolytic intermediates at the bacterial inner membrane surface.

### PgpA deletion enhances glycolytic flux and bacterial growth

GAP and G3P are important intracellular metabolites. GAP is generated from the breakdown of fructose-1,6-bisphosphate, feeding into the tricarboxylic acid (TCA) cycle via pyruvate (Fig. 1A). G3P is produced from glycerol metabolism, and it can be converted into DHAP for glycolysis (*27*). Therefore, we hypothesized that PgpA controls the levels of these two intermediates to modulate glycolytic flux.

**Fig. 2.**
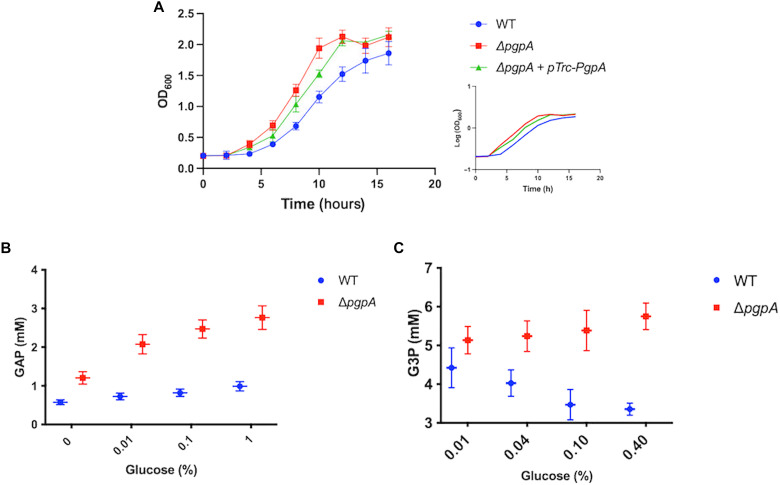
PgpA regulates *E. coli* growth and intracellular GAP/G3P levels. (**A**) Growth curves [optical density at 600 nm (OD_600_)] of *E. coli* W3110 wild type (WT), Δ*pgpA* mutant, and Δ*pgpA* complemented with plasmid-expressed PgpA under the control of a trc promoter (pTrc-PgpA). IPTG (1 mM) was added to induce protein expression. Strains were grown in M9 minimal medium containing 0.4% glycerol at 37°C with shaking. Δ*pgpA* cells exhibited enhanced growth relative to WT, which was restored by plasmid complementation. Data are shown on a linear scale as means ± SD (*n* = 3). Inset: The same data plotted on a semilogarithmic scale (log_10_ OD_600_) to facilitate comparison of exponential growth rates. (**B** and **C**) Intracellular levels of glyceraldehyde-3-phosphate (GAP) (B) and glycerol-3-phosphate (G3P) (C) were measured from total metabolite extracts of WT and Δ*pgpA* cells. Cells were cultured in M9 medium supplemented with glucose at the indicated concentrations for 6 hours at 37°C. Metabolite concentrations were quantified using colorimetric assays and then normalized to cell number and estimated average *E. coli* cell volume. Data are presented as means ± SD (*n* = 3).

To test this hypothesis, we analyzed the growth of *E. coli* strains lacking PgpA (Δ*pgpA*) in M9 medium supplemented with 0.4% glycerol. The Δ*pgpA* strain exhibited an altered growth profile, entering the exponential phase ~2 hours earlier than wild type (Fig. 2A). The exponential growth rate, reflected by the slope of the growth curve, was comparable to that of wild type, indicating that PgpA primarily modulates the transition into the exponential growth rather than the rate of proliferation. A similar growth acceleration was observed when glucose was used as the carbon source (fig. S1), indicating a general role of PgpA in central metabolic regulation. This phenotype was noticeably reversed by plasmid-based expression of PgpA (pTrc-PgpA), confirming that the effect of Δ*pgpA* results directly from *pgpA* deletion (Fig. 2A). Notably, no growth differences were observed in Δ*pgpB* or Δ*pgpC* strains (fig. S1), indicating that the phenotype is specific to PgpA and is likely to be independent of its role in PG biosynthesis.

To further investigate metabolic alterations in Δ*pgpA* cells, we quantified intracellular GAP and G3P levels using colorimetric assays, respectively. Both metabolites were substantially elevated in Δ*pgpA* cells compared to wild type (Fig. 2, B and C). In wild-type *E. coli*, GAP levels remained stable (~1 mM) across increasing glucose concentrations, whereas Δ*pgpA* cells showed progressively higher GAP levels with increased glucose availability. G3P levels also increased in Δ*pgpA*, although to a lesser extent than GAP; the difference between Δ*pgpA* and wild type became more pronounced at higher glucose concentrations. These results indicate that PgpA reduces the accumulation of GAP and G3P, thereby modulating glycolytic flux.

### Redox regulation of PgpA via disulfide cross-linking

Metabolic flux is tightly regulated in bacterial cells, but how PgpA is involved in metabolic regulation remains unclear. To explore any regulatory mechanism of PgpA in this process, we characterized its G3P phosphatase activity under varied conditions in vitro.

**Fig. 3.**
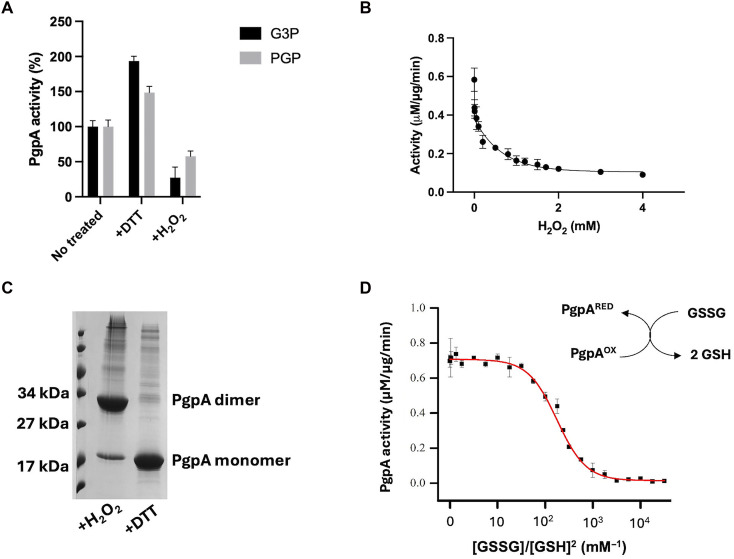
Redox regulation of PgpA activity and dimerization via disulfide bond formation. (**A**) PgpA activity was inhibited by hydrogen peroxide (H_2_O_2_) treatment, which was restored by subsequent dithiothreitol (DTT) reduction. Reactions (100 μl) containing 1 μg of purified PgpA protein and 5 μg of G3P or 40 μg of PGP were incubated at room temperature for 5 min in the absence or presence of 2 mM H_2_O_2_ or 10 mM DTT. Activities were background subtracted and normalized to the individual untreated condition, which was set to 100%. Data are presented as means ± SD (*n* = 3). (**B**) PgpA activity decreases in a dose-dependent manner with increasing concentrations of H_2_O_2_, indicating redox sensitivity. (**C**) SDS–polyacrylamide gel electrophoresis analysis of PgpA under nonreducing conditions shows dimer formation upon H_2_O_2_ treatment, which is reversed to the monomeric form by DTT. Bands corresponding to the ~34 kDa dimer and ~17 kDa monomer are indicated. (**D**) Thiol-disulfide exchange assays were performed with PgpA in the presence of varying GSH:GSSG ratios. The redox midpoint potential was estimated by nonlinear regression. Inset: Schematic illustrating reversible oxidation of PgpA thiols. Data are presented as means ± SD (*n* = 3).

**Fig. 4.**
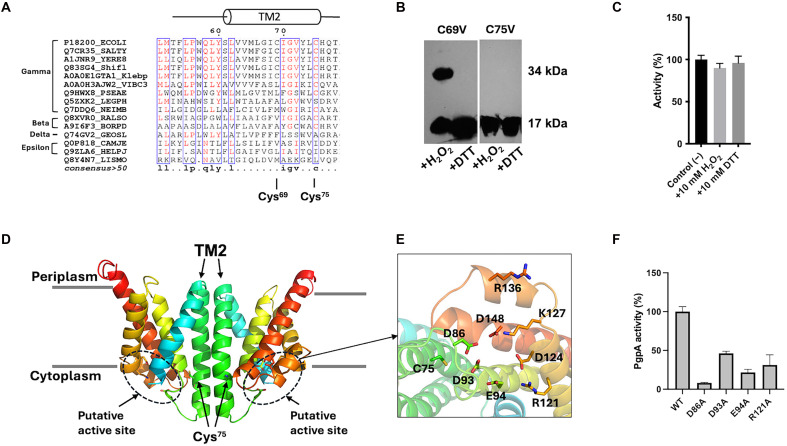
Redox regulation of PgpA mediated by the cysteine residue Cys^75^. (**A**) Multiple sequence alignment of PgpA homologs from representative Gram-negative bacteria (annotated by UniProt accession numbers) reveals that Cys^75^ is conserved in γ- and β-proteobacteria, whereas Cys^69^ is less conserved. (**B**) Immunoblot analysis of PgpA C69V and C75V mutants expressed in *E. coli* BL21(DE3) and treated with 10 mM H_2_O_2_ or 2 mM DTT. C75V prevents dimer formation under oxidative conditions. His-tagged proteins were detected using anti-His antibody. (**C**) Phosphatase activity (%) of purified PgpA-C75V protein in the presence or absence of H_2_O_2_ or DTT compared to the untreated control. The assays were conducted using 1 μg of purified proteins and 5 μg of G3P for 5 min. Activity was not affected by redox treatments, indicating that Cys^75^ is required for redox regulation. (**D**) Cartoon representation of the predicted PgpA homodimer structural model. The two protomers interact through TM2 helices, with conserved Cys^75^ residues positioned near the membrane-cytoplasm interface. (**E**) Close-up view of the putative catalytic site, showing Cys^75^ and nearby charged residues (sticks) in a hydrophilic pocket. (**F**) G3P phosphatase activity (%) of PgpA mutants at the putative catalytic residues, including D86A, D93A, E94A, and R121A, compared to wild type. Reactions were conducted using 1 μg of purified PgpA proteins and 5 μg G3P for 5 min. All data are presented as means ± SD (*n* = 3).

We found that PgpA activity was strongly inhibited by oxidative conditions, and it was reduced by ~80% upon exposure to 2 mM H_2_O_2_ (Fig. 3A), with a half-maximal inhibitory concentration of <1 μM (Fig. 3B). This inhibition was fully reversible upon treatment with 10 mM dithiothreitol (DTT) (Fig. 3A), indicating that the regulation is mediated by a reversible redox mechanism. Further analysis revealed that this redox regulation involves disulfide bond formation between PgpA monomers. SDS–polyacrylamide gel electrophoresis under nonreducing conditions showed that purified PgpA protein treated with H_2_O_2_ forms an oxidized dimer (~34 kDa), which reverts to a monomer (~17 kDa) upon DTT treatment (Fig. 4C).

Although H_2_O_2_ is commonly associated with bacterial redox regulation, *E. coli* primarily buffers its redox state through the dynamic equilibrium between reduced (GSH) and oxidized (GSSG) glutathione (*28*, *29*). We therefore examined PgpA activity using a glutathione thiol-exchange assay that also allows calculation of its redox potential. PgpA activity declined progressively as the redox environment shifted toward oxidative conditions (Fig. 3D), with a calculated midpoint redox potential (*E*_0_′) of −237 ± 5 mV, aligning with the physiological redox range of *E. coli* (−200 to −300 mV) (*30*). Together, these results reveal that PgpA activity is modulated by intracellular redox state via a disulfide-mediated dimerization mechanism.

We next examined whether PgpA activity toward its canonical lipid substrate PGP is also subject to redox regulation. Similar to the glycolytic substrate, PGP phosphatase activity was inhibited under oxidizing conditions and restored under reducing conditions (Fig. 3A). However, the magnitude of redox regulation was noticeably weaker than that observed for G3P. Oxidizing conditions reduced PGP phosphatase activity to ~60% of the reduced-state level, while a substantially basal activity (~40%) was retained. These results indicate that redox regulation preferentially targets PgpA’s glycolytic phosphatase activity rather than its lipid phosphatase function.

### Disulfide cross-linking of PgpA dimer at Cys^75^

PgpA contains two cysteine residues, Cys^69^ and Cys^75^, located within the predicted transmembrane helix 2 (Fig. 4A). We predicted these two cysteine residues as potential sites for redox regulation. To identify the residue responsible for redox sensitivity, each cysteine was individually substituted with valine to abolish thiol reactivity while preserving the hydrophobic character of TM2. The mutation of C69V did not affect the redox-dependent dimerization of PgpA. In contrast, the Cys^75^ mutant (C75V) remained monomeric under both oxidizing and reducing conditions (Fig. 4B), indicating that Cys^75^ is the chemical determinant for disulfide-mediated dimer formation. Functionally, the C75V mutant exhibited constitutive phosphatase activity regardless of the redox environment (Fig. 4C), confirming that Cys^75^ is the critical residue mediating redox-dependent regulation of PgpA activity. It is worth noting that Cys^75^ is highly conserved in γ-proteobacteria and broadly conserved in β-proteobacteria, while Cys^69^ is only sporadically present, based on sequence alignment of 316 PgpA orthologs (Fig. 4A and data S1). Therefore, the redox-regulatory mechanism involving Cys^75^ may be present in many Gram-negative bacteria.

To gain further insights into this redox regulatory mechanism, we generated a structural model of the PgpA dimer using AlphaFold 3 (fig. S2) (*31*). The model reveals that each PgpA molecule comprises five transmembrane helices (TM1-5), with dimerization primarily occurring through TM2-TM2 interactions (Fig. 4D). Cys^75^ is localized within TM2 on the cytoplasmic side of the membrane, positioned to enable disulfide cross-linking between adjacent monomers. The model also identified a putative active site composed of several conserved charged residues adjacent to Cys^75^ (Fig. 4E). Alanine substitutions at these residues within this region, including D86, D93, E94, and R121, respectively, abolished or substantially reduced enzymatic activity (Fig. 4F), validating this predicted catalytic site. Based on the spatial proximity of Cys^75^ to the active site, we hypothesize that disulfide cross-linking induces local conformational changes that allosterically inactivate the enzyme.

### Redox regulation of PgpA links glycolysis to metabolic adaptation

**Fig. 5.**
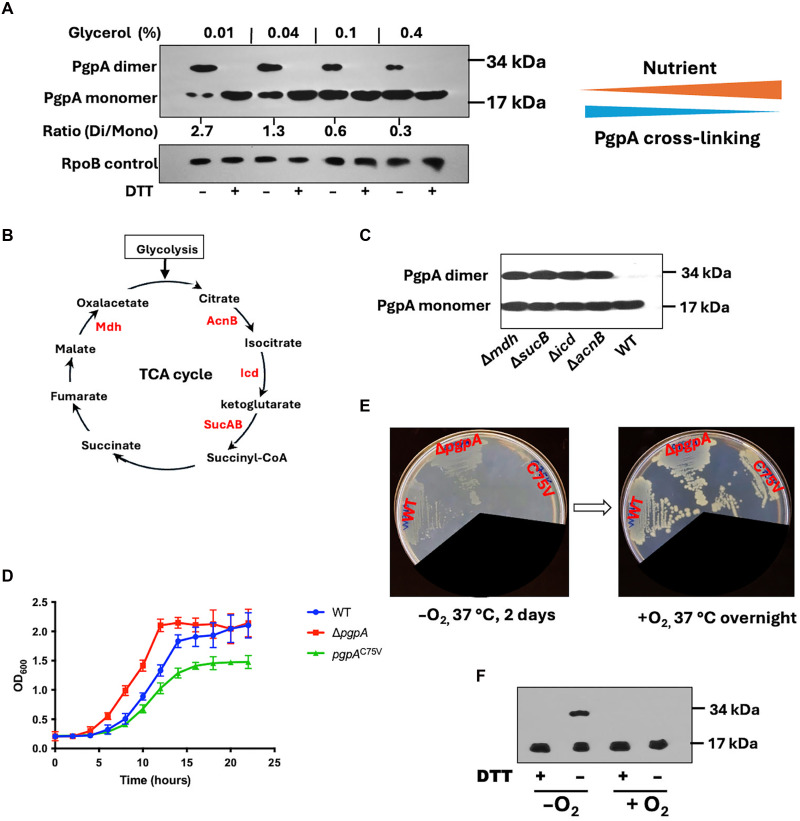
Redox regulation of PgpA is essential for metabolic adaptation and redox homeostasis. (**A**) Immunoblot analysis of His-tagged PgpA (pTrc-PgpA) expressed in *E. coli* W3110 wild-type strain grown in M9 minimal medium with increasing glycerol concentrations (0.01 to 0.4%) and induced with 1 mM IPTG for 6 hours. Cells were treated with iodoacetamide before being analyzed with or without 10 mM DTT treatment. RpoB was used as a loading control. The ratio of PgpA dimer versus monomer (Di/Mono) was quantified by densitometric analysis using ImageJ. (**B**) Schematic of the TCA cycle highlighting four redox-related enzymes (Mdh, AcnB, Icd, and SucB) whose deletions were used to assess effects on PgpA redox state. CoA, coenzyme A. (**C**) Immunoblot showing the redox state of His-tagged PgpA expressed in *E. coli* Keio knockout TCA-cycle mutants (Δ*mdh*, Δ*acnB*, Δ*icd*, and Δ*sucB*) and the parent strain BW25113. Cells were grown in M9 medium with 0.4% glycerol and induced with 1 mM IPTG for 6 hours. Samples were loaded at similar protein concentrations. (**D**) Growth curves of W3110 WT, Δ*pgpA*, and *pgpA-C75V* knock-in strains in M9 medium containing 0.4% glycerol at 37°C. Data are presented as means ± SD (*n* = 3). (**E**) Anaerobic growth phenotypes of *E. coli* W3110 WT, Δ*pgpA*, and *pgpA-C75V* strains on an LB agar plate. The plate was incubated in an anaerobic chamber for 2 days at 37°C (left) and then grown aerobically at 37°C overnight (right). (**F**) Immunoblot analysis of His-tagged PgpA expressed in *E. coli* W3110 cells grown under anaerobic (−O_2_) or aerobic (+O_2_) conditions. DTT treatment confirms that the dimeric band corresponds to disulfide-linked PgpA.

To investigate the role of PgpA disulfide cross-linking in metabolic regulation, we analyzed the redox state of PgpA expressed in *E. coli* W3110 wild type under varying carbon nutrient conditions. To preserve the in vivo redox state of PgpA, cells were treated with 10 mM membrane-permeable iodoacetamide immediately before lysis to alkylate free cysteines and prevent postlysis thiol exchange. Immunoblot analysis revealed that, under low-carbon conditions (0.01% glycerol), PgpA predominantly existed in a disulfide–cross-linked dimer (oxidized form). As glycerol concentrations increased, a shift to a monomeric (reduced) form was observed (Fig. 5A). These findings indicate that *E. coli* modulates the redox state and thus the activity of PgpA in response to carbon nutrient availability.

We next explored how PgpA responds to disruptions in central metabolism by analyzing its redox state in *E. coli* Keio knockout strains lacking key TCA cycle enzymes (Δ*acnB*, Δ*icdA*, Δ*sucB*, and Δ*mdh*) (Fig. 5B) (*32*), which are known to substantially impair bacterial metabolism and growth (*33*, *34*). In all TCA cycle mutants, PgpA persisted in the oxidized, disulfide-linked dimeric form even under high (0.4%) glycerol conditions (Fig. 5C), suggesting that diminished metabolic flux promotes PgpA oxidation, potentially through a redox-sensitive feedback inhibition mechanism.

To further examine the physiological roles of PgpA redox regulation, we generated a chromosomal knock-in *E. coli* mutant (*pgpA*-*C75V*) that mimics a constitutively active, redox-insensitive form of the enzyme. In contrast to the Δ*pgpA* strain, which enters exponential growth prematurely, the *pgpA*-*C75V* strain demonstrated a delayed transition into the exponential phase in nutrient-rich conditions (Fig. 5D). This growth defect was further exacerbated under anaerobic conditions, where energy metabolism is limited even in the LB medium: The *pgpA*-*C75V* strain failed to grow after 2 days in the absence of oxygen (Fig. 5E and fig. S2). Notably, growth was immediately restored upon reexposure to aerobic conditions, suggesting that the cells remained viable but were metabolically suppressed.

We further examined the redox state of PgpA using the alkylation method described above. Immunoblot analysis revealed that PgpA predominantly existed in the monomeric form when cells were grown aerobically in LB medium but accumulated as a disulfide-linked dimer under anaerobic conditions (Fig. 5F). These findings underscore the essential role of PgpA redox regulation in mediating metabolic switching in response to environmental oxygen availability.

### PgpA mutations interrupt the bacterial central metabolic balance

**Fig. 6.**
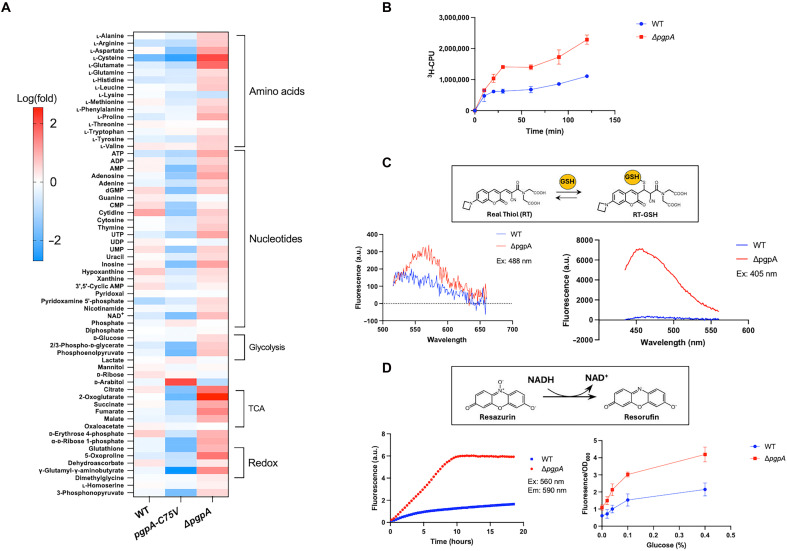
PgpA contributes to metabolic regulation and cellular redox homeostasis. (**A**) Heatmap showing changes in intracellular metabolite levels (log_10_-transformed values) between 8 and 4 hours in *E. coli* wild type W3110 (WT), Δ*pgpA*, and *pgpA-C75V* strains. Metabolites are grouped into categories: amino acids, nucleotides, glycolysis, TCA cycle, and redox-related intermediates. ADP, adenosine 5′-diphosphate; AMP, adenosine 5′-monophosphate; CMP, cytidine 5′-monophosphate; dGMP, deoxyguanosine monophosphate; UTP, uridine 5′-triphosphate; UDP, uridine 5′-diphosphate; UMP, uridine 5′-monophosphate. (**B**) Glucose uptake time course in WT and Δ*pgpA* strains, measured using radiolabeled, nonmetabolizable glucose analog 3-0-[methyl-d-1-^3^H]-glucose. Δ*pgpA* cells exhibit increased glucose uptake compared to WT. (**C**) In vivo glutathione fluorescence assays in WT and Δ*pgpA* using Real Thiol (RT). Insert: Chemical structure of RT, a cell-permeable fluorescent probe specific for GSH. Fluorescence emission spectra measured using excitation at 488 nm (left) or 405 nm (right) show elevated GSH levels in Δ*pgpA* cells. (**D**) In vivo redox status measured using resazurin, a NADH-sensitive probe that fluoresces upon reduction by NADH, in WT and Δ*pgpA* strains. Insert: Chemical structures of resazurin and its reduced product, resorufin. Left: Real-time monitoring of resorufin fluorescence during growth in M9 medium with 0.4% glucose [excitation (Ex), 560 nm; and emission (Em), 590 nm]. Right: Endpoint fluorescence intensity normalized to OD_600_ in cells exposed to increasing glucose concentrations (0 to 0.4%). Δ*pgpA* cells show enhanced resazurin reduction, consistent with a more reduced intracellular redox state. Data are presented as means ± SD (*n* = 3). a.u., arbitrary units.

The redox regulation of PgpA suggests a link between glycolysis and cellular redox homeostasis. To investigate this connection, we conducted untargeted metabolomic profiling of *E. coli* W3110 wild-type, Δ*pgpA*, and *pgpA*-*C75V* strains. Given that PgpA-dependent growth phenotypes emerged during the early exponential phase (Fig. 5D), intracellular metabolite levels were compared between 4 and 8 hours postinoculation. Wild-type cells exhibited relatively stable metabolic profiles during this period, whereas both Δ*pgpA* and *pgpA*-*C75V* strains showed broad and opposing changes across glycolysis, the TCA cycle, amino acid metabolism, nucleotide metabolism, and redox pathways (Fig. 6A and data S2).

Specifically, Δ*pgpA* cells accumulated key intermediates, whereas *pgpA*-*C75V* mutants displayed widespread depletion. For example, pyruvate levels increased 6.9-fold (*P* = 0.025) in Δ*pgpA* cells but decreased 3-fold (*P* = 0.002) in *pgpA*-*C75V*. Similarly, TCA intermediates such as citrate and 2-oxoglutarate increased 43-fold (*P* = 0.03) and 356-fold (*P* = 0.007) in Δ*pgpA* but decreased 37-fold (*P* = 4.9 × 10^−6^) and 71-fold (*P* = 0.003), respectively, in *pgpA-C75V*. This pattern was also observed in amino acid pools and nucleotide triphosphates. For instance, ATP levels increased 11-fold (*P* = 0.04) in Δ*pgpA* but dropped 10-fold (*P* = 0.0004) in *pgpA*-*C75V*. These findings highlight PgpA as a central metabolic regulator by tuning glycolytic flux and downstream energy and biosynthetic pathways during early exponential growth.

### PgpA deletion enhances glucose uptake via the PTS system

Bacterial glycolysis is tightly regulated by glucose availability (*35*, *36*). Given the accumulation of glycolytic and TCA intermediates in Δ*pgpA* cells, we hypothesized that PgpA deletion enhances glucose uptake. In *E. coli*, glucose is primarily imported via the phosphoenolpyruvate (PEP)–dependent phosphotransferase system (PTS) (*37*). PEP levels were decreased sixfold (*P* = 1.04 × 10^−5^) in *pgpA*-*C75V* mutant compared to only twofold (*P* = 0.03) in wild-type (Fig. 6A), suggesting that PgpA activity influences PTS-mediated glucose transport.

To test this directly, we measured glucose uptake using the nonmetabolizable glucose analog 3-O-[methyl-d-1-^**3**^H] glucose. Δ*pgpA* cells exhibited a twofold increase in glucose uptake compared to wild type (Fig. 6B), supporting the conclusion that PgpA negatively regulates glucose transport and glycolysis during early exponential growth.

### PgpA regulates redox balance via glutathione biosynthesis

A key remaining question is how *E. coli* adjusts its redox state to control PgpA activity in response to glycolytic flux. Metabolomic profiles revealed that PgpA influences glutathione biosynthesis (Fig. 6A and data S2). For instance, the levels of cysteine, a key GSH precursor, rose by 122-fold (*P* = 0.01) in Δ*pgpA* but dropped by 250-fold (*P* = 1.6 × 10^−4^) in *pgpA*-*C75V* and were only modestly reduced (6-fold, *P* = 0.02) in wild type. Additionally, γ-glutamyl-γ-aminobutyrate, a by-product of glutathione turnover, increased 24-fold (*P* = 0.05) in Δ*pgpA* and decreased ~500-fold (*P* = 1.5 × 10^−6^) in *pgpA*-*C75V*, consistent with enhanced glutathione production.

To validate these findings, we directly quantified intracellular glutathione in living *E. coli* using the Real Thiol (RT) fluorescence assay (Fig. 6C). RT is a cell-permeable fluorescent probe that selectively reacts with GSH, enabling real-time quantification of intracellular GSH levels (*38*). Δ*pgpA* cells exhibited markedly higher fluorescence than the same amount of wild type, corresponding to intracellular GSH concentrations of 3.9 ± 0.5 mM in Δ*pgpA* versus 1.04 ± 0.11 mM in wild type. This more reduced redox state was further confirmed using resazurin, a redox-sensitive dye, which showed accelerated reduction kinetics in Δ*pgpA* cells (Fig. 6D). Together, these results indicate that PgpA inactivation promotes glycolysis-driven glutathione biosynthesis, thereby shifting the intracellular redox balance toward a more reduced state. These findings suggest that PgpA serves as a critical regulatory link between glycolysis and intracellular redox homeostasis.

## DISCUSSION

Our study identifies PgpA as a redox-sensitive phosphatase that plays a central role in modulating bacterial energy metabolism. Under reducing conditions, PgpA remains in its monomeric, active form on the cytoplasmic membrane surface of the inner membrane, where it hydrolyzes glycolytic intermediates GAP and G3P to limit glycolytic flux (Figs. 1, C and D, and 3A). When intracellular redox conditions shift toward an oxidized state, PgpA dimerizes through disulfide bond formation at Cys^75^, resulting in enzymatic inactivation through dimerization (Figs. 3C and 4B). This redox-dependent dimerization functions as a metabolic switch, enabling *E. coli* to dynamically regulate glycolytic output in response to environmental changes (Fig. 5, A and F).

Loss of PgpA function (Δ*pgpA*) leads to the accumulation of GAP and G3P, increased glucose uptake, and enhanced activity of glycolysis and TCA cycle, promoting biosynthesis of amino acids, ATP, and NADH (Figs. 2, B and C, and 6, A, B, and D). Conversely, constitutive activation of PgpA (*pgpA*-*C75V*) suppresses metabolic activity and impairs growth under nutrient-limited or anaerobic conditions (Figs. 5, D and E, and 6A). These results indicate PgpA as a key regulator of metabolic balance across fluctuating environments.

Our data support a functional link between glycolysis and redox homeostasis mediated by PgpA. In Δ*pgpA* cells, elevated glycolytic activity stimulates glutathione biosynthesis, shifting the intracellular redox balance toward a more reduced state (Fig. 6, A, C, and D). In contrast, *pgpA*-*C75V* cells exhibit depleted glutathione precursors and impaired redox buffering capacity, resulting in metabolic suppression (Fig. 6A). These findings position PgpA as a redox-sensitive metabolic switch that connects energy flux with redox regulation.

**Fig. 7.**
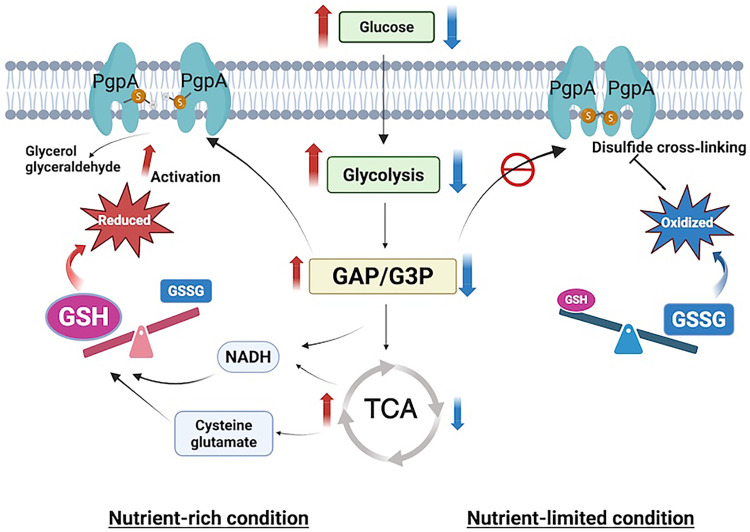
Redox-sensitive negative feedback regulation of glycolysis by PgpA in response to nutrient conditions. A schematic model illustrates the dynamic regulation of glycolytic flux and redox homeostasis mediated by the membrane-associated phosphatase PgpA in *E. coli*. Left (Nutrient-rich conditions): PgpA exists in a reduced, monomeric, and active state. It dephosphorylates glycolytic intermediates GAP and G3P, thereby limiting glycolytic flux, reducing glucose uptake, and maintaining metabolic homeostasis. Right (Nutrient-limited conditions): PgpA forms a disulfide-linked dimer via Cys^75^, resulting in enzymatic inactivation. This leads to the accumulation of GAP and G3P, which facilitates glycolysis, increases glucose uptake, and promotes glutathione (GSH) biosynthesis. Elevated GSH restores a reducing intracellular environment, reactivating PgpA and completing a negative feedback loop. This regulation enables *E. coli* to adaptively tune glycolysis based on both redox balance and nutrient availability.

These results support a metabolic regulation model mediated by PgpA (Fig. 7). Under nutrient-rich conditions, active glycolysis and TCA cycle activity facilitate biosynthesis and glutathione production, maintaining a reduced intracellular state that sustains PgpA activity to hydrolyze glycolytic intermediates, thereby limiting excess metabolic flux and preserving homeostasis. Under nutrient-poor or oxidative conditions, reduced flux promotes PgpA inactivation via disulfide-mediated dimerization, relieving the constraint on glycolysis. This enhances energy production and stimulates glutathione synthesis, ultimately restoring a reduced state that reactivates PgpA. Through this feedback loop, PgpA enables *E. coli* to fine-tune its metabolic and redox states in real time.

Our results also unmask PgpA as a moonlighting enzyme with dual roles in phospholipid metabolism and glycolytic regulation. The positioning of its active site on the cytoplasmic surface is strategically designed to integrate cytoplasmic metabolism and membrane-associated processes (Fig. 4D), which allows it to directly access its cytoplasmic substrates, GAP, and G3P, as well as membrane-resident phospholipids (Fig. 1, C and D). Moreover, the positioning of Cys^75^ on the cytoplasmic face situates PgpA near redox-active species such as quinones and glutathione pools, consistent with its role as a redox sensor. This localization may also allow indirect regulation of the PTS system, as suggested by increased glucose uptake in Δ*pgpA* cells (Fig. 6B), potentially mediated by elevated PEP levels (Fig. 6A). By residing at this metabolic crossroads, PgpA integrates glycolytic control with redox regulation and membrane lipid metabolism, ensuring a coordinated response to environmental cues.

Our study raised a question of how PgpA performs its moonlighting function. Our results indicate that redox regulation of PgpA preferentially targets its activity toward glycolytic substrates rather than its canonical lipid substrate PGP (Fig. 3A), thereby preserving lipid metabolism. In addition to PgpA, *E. coli* encodes two other PGP phosphatases, PgpB and PgpC (*23*). These three enzymes are encoded in distinct operons and share no sequence homology, suggesting functional divergence (*25*). Although the specific physiological role of PgpC remains unclear, PgpB has been shown to participate in peptidoglycan biosynthesis through dephosphorylation of undecaprenyl pyrophosphate (C55-PP) (*39*), and its structure provides no evidence for redox-dependent regulation (*40*). The presence of PgpB and PgpC allows phospholipid biosynthesis to remain uninterrupted as maintenance of any single isoform is sufficient to sustain PG production at near–wild-type levels (*23*). The functional redundancy safeguards membrane lipid homeostasis, while PgpA has evolved specialized redox-sensitive regulation to selectively modulate glycolytic flux without compromising essential lipid metabolism.

PgpA is broadly present in Gram-negative bacteria. The conservation of the residue Cys^75^ across γ- and β-proteobacteria (Fig. 4A and data S1) suggests that its redox-sensing mechanism may have broad physiological relevance in these organisms. γ-Proteobacteria represent the largest subclass of Gram-negative microorganisms and include many prominent human pathogens such as *P. aeruginosa*, *Salmonella enterica*, and *Edwardsiella tarda*. It has been reported that glucose supplementation in *E. tarda* stimulates bacterial central metabolism, consequently alleviating antibiotic resistance by enhancing drug uptake (*41*), a process that may be regulated by PgpA. Future studies in these bacteria will confirm whether PgpA functions as a conserved redox-mediated metabolic regulator and may help identify the potential role of PgpA in modulating antibiotic susceptibility.

In addition, our finding that constitutive activation of PgpA (C75V) impairs *E. coli* growth under anaerobic conditions (Fig. 5E and fig. S2) suggests that maintaining threshold levels of glycolysis is critical for survival in low-oxygen environments such as biofilms or abscesses. This raises the possibility that pharmacological stabilization of PgpA’s active form, e.g., by preventing Cys^75^ cross-linking, could disrupt anaerobic persistence and offer a strategy to suppress bacterial growth under those challenging conditions.

The redox regulatory mechanism of PgpA described here is likely specific to microorganisms. Although a PGP phosphatase, PTPMT1, has been identified in mammalian mitochondria (*42*), it shares no sequence homology with PgpA and lacks features consistent with a disulfide-based redox switch. To our knowledge, this study reports a unique redox regulatory mechanism operating at the bacterial inner membrane. Redox regulation is a fundamental principle for coordinating metabolic activity with the intracellular redox state. In both prokaryotic and eukaryotic cells, energy metabolism is extensively controlled by redox-dependent processes, and numerous enzymes and transcriptional factors, predominantly cytosolic proteins, are regulated through reversible cysteine modifications. Future studies may determine whether similar principles operate to regulate the functions of membrane proteins in response to intracellular redox balance.

Despite some open questions, our work reveals a previously unrecognized redox-sensing mechanism that links glycolysis and redox homeostasis at the bacterial inner membrane. This discovery provides a framework for understanding how bacteria integrate metabolic and redox signals to maintain homeostasis and adapt to environmental changes.

## MATERIALS AND METHODS

### Bacterial strains and growth conditions

*E. coli* strains and plasmids used in this study are listed in table S1. pTrc-PgpA was generated by inserting the *pgpA* gene of *E. coli* into the pDSW195 vector (*43*) to allow the expression of N-terminal His-tagged PgpA under an isopropyl-β-d-thiogalactopyranoside (IPTG)–induced P_trc_ promoter. *E. coli* wild-type or mutant strains were cultured in LB [tryptone (10 g/liter), yeast extract (5 g/liter), and NaCl (10 g/liter)] or M9 minimal medium [Na_2_HPO_4_ (6 g/liter), KH_2_PO_4_ (3 g/liter), NaCl (0.5 g/liter), and NH_4_Cl (1 g/liter)] supplemented with 1 mM MgCl_2_, 0.1 mM CaCl_2_, 0.001% thiamine, and the indicated concentrations of glucose or glycerol as carbon source.

For mutant analysis and metabolite extraction, a single colony was picked from an LB agar plate and cultured overnight in LB at 37°C. The overnight culture was diluted 1:100 into M9 medium supplemented with 0.4% glucose or glycerol and grown overnight at 37°C. The following day, cells were pelleted, washed three times with M9 medium, and resuspended in fresh M9 medium to an optical density at 600 nm (OD_600_) of 0.2. Cultures were then grown at 37°C with shaking for 6 hours or as indicated.

Anaerobic experiments were performed in an anaerobic chamber (85% N_2_, 10% CO_2_, and 5% H_2_). LB was dispensed into Bellco flasks and equilibrated overnight inside the anaerobic chamber. A 1:100 inoculum from an LB preculture was added to the preequilibrated medium, and flasks were sealed and incubated at 37°C. Cell density was monitored at specified time points using a Klett meter. For aerobic recovery, flask caps were loosened the following day, and cultures were transferred to a 37°C shaking incubator for continued growth.

### Construction of mutant strains

Δ*pgpA* and *pgpA*-*C75V* strains were generated in the background of *E. coli* wild-type W3110 strain using standard λ-Red homologous recombination techniques (*44*, *45*). Primer sequences and sequencing results are listed in table S2. pTrc-PgpA was generated using the plasmid pDSW195.

For chromosomal knockout of *pgpA*, a linearized pKD3 plasmid was used as a template to generate a polymerase chain reaction (PCR) product with primers pgpA KO forward and pgpA KO reverse. The resulting DNA cassette contained a chloramphenicol resistance gene flanked by homology arms corresponding to the 5′ and 3′ terminal regions of *pgpA*. This cassette was introduced into W3110 cells carrying the pKD46 plasmid via electroporation. The addition of 10 mM l-arabinose induced expression of the λ-Red recombinase. Cells were incubated at 32°C in LB containing ampicillin and l-arabinose for 2 hours and then plated on LB agar with chloramphenicol and incubated overnight at 30°C. Gene replacement was verified by colony PCR. To eliminate the pKD46 plasmid, positive transformants were grown overnight in LB medium at 37°C without antibiotics and screened for ampicillin and chloramphenicol sensitivity.

To generate the *pgpA*-*C75* knock-in strain, a two-step selection and replacement protocol was performed using the Kn-pBAD-ccdB cassette. λ-Red recombinase was induced in W3110 cells carrying the temperature-inducible pSIM6 plasmid. In the first step, the Kn-pBAD-ccdB cassette was PCR amplified with primers, pgpA [50 base pairs (bp)] and KN1 forward and pgpA (50 bp) and ccdB1 reverse, and then introduced into λ-Red–induced cells via electroporation. Cells were incubated in 2 ml of LB containing 1% glucose at 32°C for 2 hours to allow homologous recombination. Recombinants were selected on LB agar plates supplemented with ampicillin, kanamycin, and 1% glucose and incubated overnight at 30°C. Integration of the Kn-pBAD-ccdB cassette was confirmed by PCR and differential growth on LB plates containing 1% glucose or 1% arabinose.

In the second step, a PCR product encoding the *pgpA*-*C75* mutations was generated using primers, pgpA forward and pgpA reverse, and transformed into cells carrying the Kn-pBAD-ccdB cassette. After 2 hours of incubation in LB with 1% glucose at 32°C, recombinants were selected on LB agar containing ampicillin and 1% l-arabinose and incubated overnight at 37°C. Correct replacement of the selection cassette with the mutant allele was verified by PCR. The pSIM6 plasmid was removed by overnight growth at 37°C and confirmed by ampicillin sensitivity. All mutations were confirmed by PCR and Sanger sequencing.

### Protein expression and purification

PgpA wild-type and mutant proteins from *E. coli* were expressed using a T7 promoter–driven plasmid in the *E. coli* BL21(DE3) strain, following the recently described purification protocol (*25*). Site-directed mutations were generated using a modified Quick-change mutagenesis protocol (*46*).

*E. coli* BL21(DE3) cells were cultivated in an autoinduction medium (*47*) at 37°C for 3 hours. Subsequently, the culture was incubated at 18°C overnight to facilitate protein expression. Following incubation, cells were harvested and resuspended in a lysis buffer consisting of 50 mM tris-HCl (pH 8.0), 500 mM NaCl, and 10% glycerol. Cell lysis was achieved by subjecting the suspension to an EmulsiFlex-C3 homogenizer (Avestin) at 15,000 psi for three cycles.

After lysis, the suspension underwent centrifugation to clarify the lysate. The membrane fractions were then pelleted using a Ti45 rotor at 40,000 rpm for 1 hour. The resultant membrane pellet was solubilized in lysis buffer containing 1% (w/v) *n*-dodecyl-β-d-maltoside (DDM) at 4°C for 1.5 hours. The solubilized membrane fractions were subsequently loaded onto a Ni^2+^-charged NTA column (Cytiva), which had been preequilibrated with lysis buffer containing 0.05% DDM and 20 mM imidazole. Following this, the column was washed with buffer containing 40 mM imidazole, and the proteins were eluted using 400 mM imidazole. The eluted protein was buffer-exchanged into 20 mM tris-HCl (pH 7.5), 200 mM NaCl, 2 mM MgCl_2_, 4% glycerol, and 0.05% DDM using a desalting column (Cytiva). Protein concentration was determined using the Bradford protein assay (Bio-Rad).

### Preparation of inside-out vesicles

Uniformly oriented inside-out membrane vesicles were prepared using a low-pressure homogenization method (*48*). Briefly, PgpA was expressed in *E. coli* BL21(DE3) cells by adding 1 mM IPTG for 3 hours. Cells were resuspended in buffer containing 100 mM tris-HCl (pH 8.0), 250 mM NaCl, 2 mM MgCl_2_, and 2 mM DTT, and homogenized at 4000 psi for one cycle. Cellular debris was removed by centrifugation at 12,000 rpm for 15 min (SS-34 rotor), and vesicles were collected by ultracentrifugation at 40,000 rpm for 1 hour (Ti-45 rotor). Vesicles were washed and resuspended in the same buffer using a Teflon homogenizer and then flash-frozen in liquid nitrogen for storage.

### PgpA phosphatase assays

PgpA activity was measured by quantifying phosphate release using a malachite green–based colorimetric assay (*49*). The colorimetric reagent was prepared by mixing stock solutions of 34 mM ammonium molybdate (dissolved in 5 M HCl) and 2.16 mM malachite green oxalate at a 1:3 (v/v) ratio, followed by filtration to ensure clarity and consistency.

Reactions were carried out at 25°C in 100 μl of solution containing purified PgpA protein and substrates as indicated. At the indicated time, reactions were terminated by adding 200 μl of colorimetric reagent, followed by adding 30 μl of 1% polyvinyl alcohol to stabilize the color development. Absorbance was measured at 660 nm using a spectrophotometer. Free inorganic phosphate concentrations were quantified by comparison to a potassium phosphate standard curve.

Vesicle-based assays were initiated by adding 1 mM G3P or 5 mM GAP to 1 ml of vesicles (total protein of 0.2 mg/ml) at room temperature. At indicated times, the reactions were terminated by adding 5 mM EDTA and then passed through a 10-kDa molecular weight cutoff centrifugal filter to separate vesicles from free phosphate. The filtrates were measured using the malachite green assay as described above. Data were normalized by subtracting background phosphate levels measured in parallel reactions lacking enzyme or vesicles.

### Glutathione thiol exchange assay

The redox potential of PgpA was calculated by following a thiol exchange method previously described (*30*). G3P phosphatase was measured as above in the presence of varied ratios of GSH to GSSG. Redox titration data were fitted using the KaleidaGraph software to calculate the equilibrium constant *K*_eq_, representing the midpoint of the enzyme activity. This value was then used to calculate the standard state redox potential of PgpA (*E*°′_PgpA_) based on a protocol previously published and the known state redox potential of the GSH/GSSG couple (*E*°′_GSH_ = −240 mV) (*50*).

### Metabolite extraction

Bacterial metabolites from *E. coli* were extracted using a chilled methanol-based protocol (*51*). Cells were grown in M9 medium supplemented with glucose at 37°C with shaking and harvested after 6 hours of incubation for biochemical assays or after 4 or 8 hours for metabolomic study. Metabolites were extracted in three sequential steps: (i) Approximately 4 × 10^8^ cells were harvested on the basis of OD_600_ measurements and confirmed by colony-forming unit counts. Cell pellets were immediately treated with 300 μl of 100% methanol prechilled on dry ice for 15 min to rapidly quench metabolic activity. The mixture was centrifuged at 4°C, and the supernatant (first extract) was collected. (ii) The remaining pellet was resuspended in 100 μl of 80:20 methanol:water solution and subjected to sonication for 15 min at 4°C. The supernatant (second extract) was collected following centrifugation. (iii) Step 2 was repeated to obtain a third extract. All three extracts were pooled, lyophilized, and reconstituted in 100 μl of buffer [100 mM potassium phosphate and 5 mM EDTA (pH 7.5)] for subsequent biochemical and metabolomic analysis. Metabolomic analysis was performed by the University of Colorado School of Medicine Metabolomics Core.

### G3P and GAP assays

Intracellular levels of G3P and GAP were quantified using commercial colorimetric assay kits following the manufacturers’ protocols: the G3P Assay Kit (AAT Bioquest) and the GAPDH Activity Assay Kit (BioVision), respectively. For G3P quantification, 5 μl of the extracted metabolite sample was mixed with 45 μl of KPE buffer and 50 μl of the G3P assay reagent. The reaction was incubated at 25°C for 30 min in the dark. Fluorescence was measured using a microplate reader at 540-nm excitation and 590-nm emission.

For GAP measurement, 50 μl of the extracted sample was combined with 48 μl of GAPDH Assay Buffer, 2 μl of GAPDH Developer, and 0.36 units of GAPDH enzyme, yielding a total volume of 100 μl per well. The reaction was incubated at 37°C for 30 min, and absorbance was measured at 450 nm using a microplate reader. Concentrations of G3P and GAP were calculated from standard curves and normalized to cell number and an estimated *E. coli* average cell volume of 1 μm^3^.

### Glucose uptake assay

Glucose uptake was measured using the radiolabeled, nonmetabolizable glucose analog 3-0-[methyl-d-1-^3^H]-glucose. *E. coli* strains were first cultivated in M9 medium supplemented with 1% glucose at 37°C for 6 hours. Cells were harvested, washed three times with M9 minimal medium without carbon source, and resuspended to an OD_600_ of 10.

Uptake assays were initiated by adding 3-O-[methyl-d-1-^3^H]-glucose at a final radioactivity of 5 μCi/ml (from a stock solution of 1 mCi/ml), together with 2 mM unlabeled 3-O-methyl-d-glucopyranose to the cell suspension, followed by incubation at 37°C. To terminate uptake, the reaction mixture was rapidly diluted fivefold into an ice-cold M9 minimal medium. Cells were immediately filtered through Whatman GF/F glass fiber filters using a Millipore vacuum manifold, and filters were washed once with 10 ml of ice-cold M9 salt solution. Radioactivity retained on the filters was measured by liquid scintillation counting. Glucose uptake activities were normalized to cell density on the basis of OD_600_ readings.

### Glutathione RT assay

Measurement of intracellular glutathione levels in living *E. coli* was performed using RT, a cell-permeable fluorescent probe that enables real-time, quantitative detection of GSH, as previously described with minor modifications (*38*).

Approximately 10^9^ cells were harvested from M9 culture supplemented with 1% glucose and resuspended in 1 ml of fresh medium. Cells were then incubated with 1 μM RT for 30 min at 25°C with shaking. Following incubation, ~5 × 10^8^ cells were pelleted and washed three times with M9 medium to remove unbound dye, then resuspended in 1 ml of fresh M9 medium.

Fluorescence was measured using a microplate reader with dual excitation/emission settings: excitation at 405 nm/emission at 418 to 499 nm for the RT-GSH adduct and excitation at 488 nm/emission at 499 to 615 nm for free RT. The intracellular GSH concentration was determined by calculating the fluorescence intensity ratio (*I*_405_/*I*_488_) and interpolating from a standard GSH calibration curve. Final GSH levels were normalized to total cell number and an estimated average *E. coli* cell volume of 1 μm^3^.

### Bacterial fluorescence redox assays

Redox changes in *E. coli* cells were assessed using resazurin, a redox-sensitive fluorescent indicator. Overnight cultures were diluted into fresh M9 medium supplemented with 1% glucose to an OD_600_ of 0.2. Resazurin was added to a final concentration of 0.05 mM in a 96-well plate. Fluorescence was measured using a plate reader with excitation at 560 nm and emission at 590 nm. Fluorescence values were normalized to total cell number.

### Statistical analysis

Data are presented as means ± SD. Statistical analyses were performed using a two-tailed unpaired Student’s *t* test, where applicable. Differences were considered statistically significant at *P* < 0.05.
