# Supplementary material for: A redox-sensitive phosphatase regulates glycolysis as a metabolic switch in the bacterial inner membrane
Source: Sci Adv. 2026 May 6;12(19):eaea8724. doi: 10.1126/sciadv.aea8724 (PMC13148334; doi:10.1126/sciadv.aea8724)
Supplement: Supplementary file 1 — Figs. S1 to S3 Tables S1 and S2 Legends for data S1 and S2 [file sciadv.aea8724_sm.pdf]

Science Advances
AAAS

Supplementary Materials for

A redox-sensitive phosphatase regulates glycolysis as a metabolic switch in the bacterial inner membrane

Lei Zheng *et al.*

Corresponding author: Lei Zheng, lei.zheng@uth.tmc.edu

Sci. Adv. **12**, eaea8724 (2026)
DOI: 10.1126/sciadv.aea8724

The PDF file includes:

Figs. S1 to S3
Tables S1 and S2
Legends for data S1 and S2

Other Supplementary Material for this manuscript includes the following:

Data S1 and S2

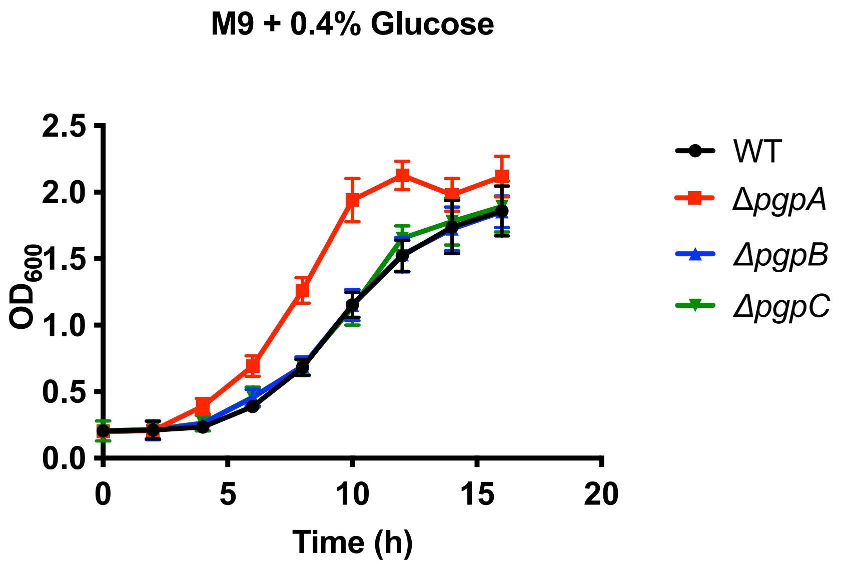

Fig. S1. Growth curves of *E. coli* W3110 wild-type (WT) and Δ*pgpA*, Δ*pgpB*, and Δ*pgpC* mutant strains.

Cells were grown in M9 minimal medium supplemented with 0.4% glucose at 37°C with shaking. Optical density at 600 nm (OD_{600}) was measured at the indicated time points. The Δ*pgpA* mutant exhibited enhanced growth compared to the WT and other PG knockout strains, including Δ*pgpB* and Δ*pgpC*. Data are presented as mean ± SD ($n = 3$).

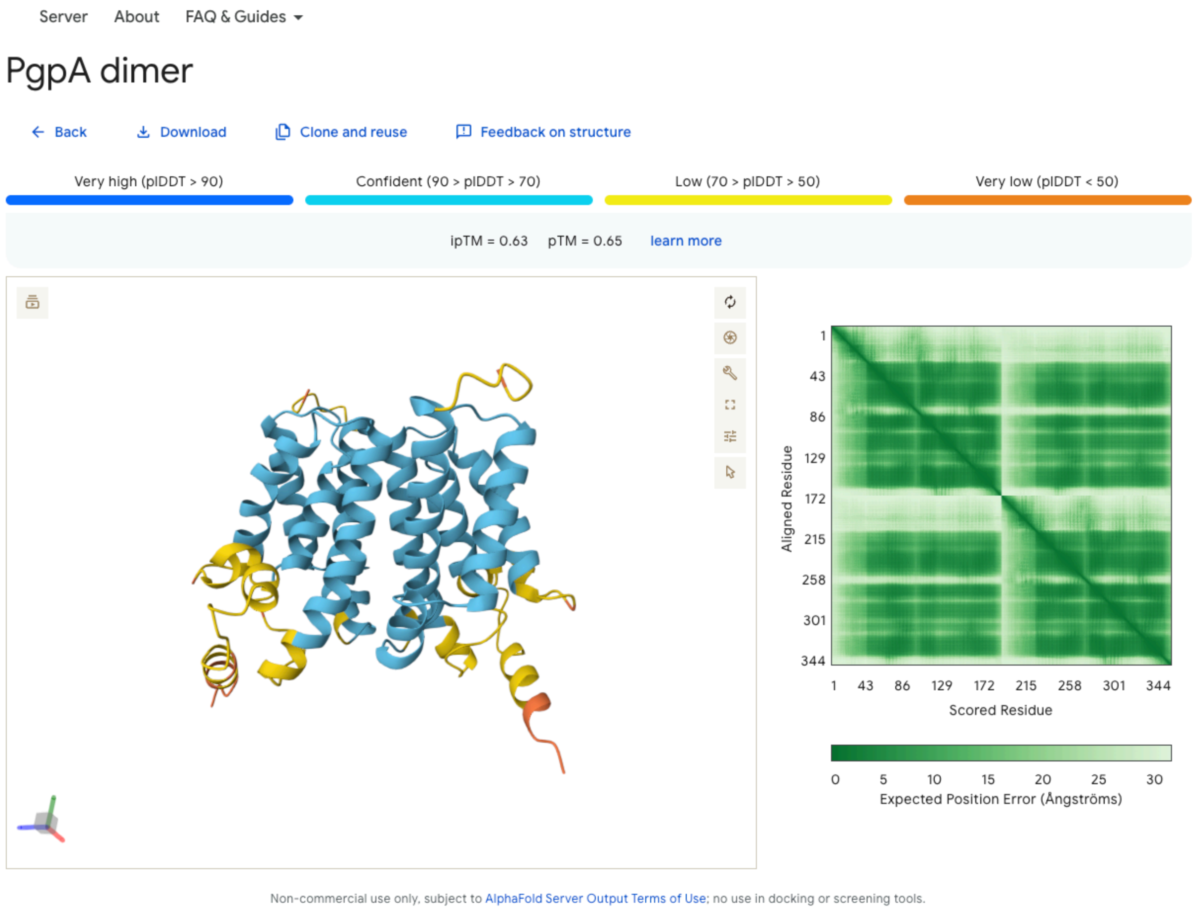

Fig. S2. Structural model of PgpA homodimer of *E. coli*.
The model was generated using AlphaFold 3 (www.alphafoldserver.com). The structure is colored by confidence score, with regions modeled at high confidence shown in blue, particularly the transmembrane domains. The predicted template modeling (pTM) score is 0.65, and the inter-chain pTM (ipTM) score is 0.63. A pTM score above 0.5 suggests that the overall fold of the predicted complex is likely to resemble the true structure.

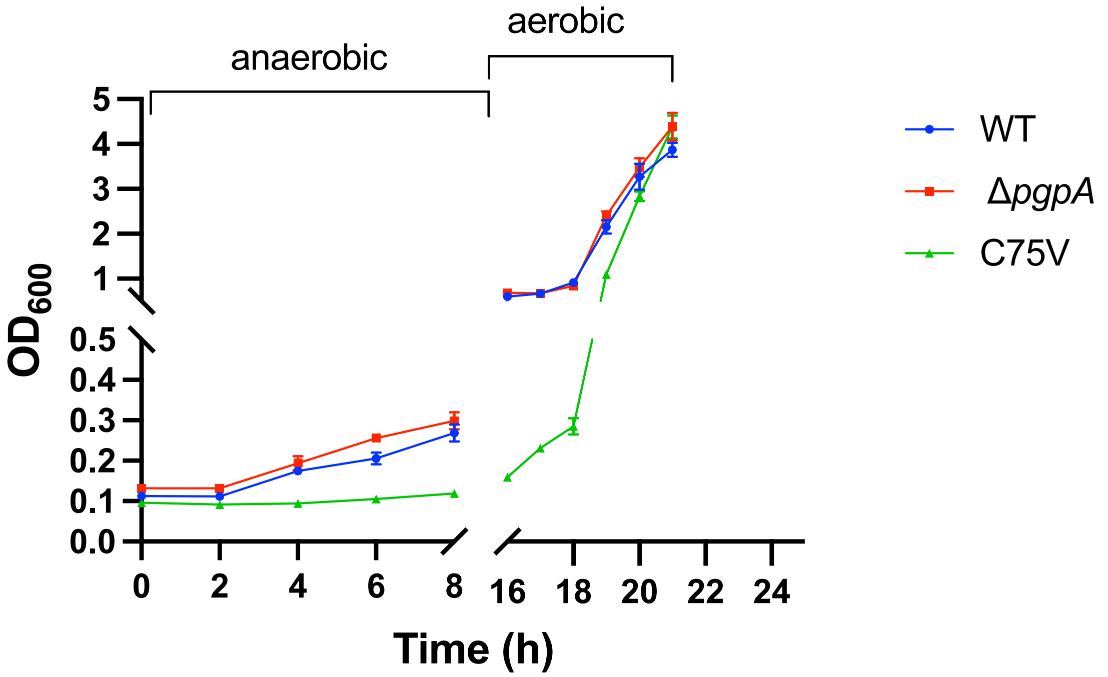

Fig. S3. The C75V mutation impairs *E. coli* growth under anaerobic conditions.
Growth curves of *E. coli* wild-type (WT), Δ*pgpA,* and *pgpA-C75V* mutant strains. Cells were grown in LB medium at 37°C for 16 hours under anaerobic conditions, followed by continued incubation under aerobic conditions at 37°C with shaking. Optical density at 600 nm (OD_{600}) was measured at the indicated time points. Data represent mean ± SD (*n* = 3).

Table S1. Key resource used in this study.

**Antibodies**		
Mouse monoclonal anti-RpoB	Santa Cruz Biotechnology	Cat# SC-101613; RRID: AB_1129055
Mouse monoclonal anti-His	LifeTein, LLC	Cat# LT0426
**Bacterial strains**		
BL21(DE3)	New England Biolab	
W3110	ATCC	
W3110 *ΔpgpA*	This study	
W3110 Δ*pgpB*	(23)	
W3110 Δ*pgpC*	(23)	
W3110 *pgpA-C75V*	This study	
BW25113	(32)	
BW25113 Δ*icd*	(32)	
BW25113 Δ*icd*	(32)	
BW25113 Δ*rmf*	(32)	
BW25113 Δ*icd*	(32)	
**Plasmids**		
pET-PgpA	(25)	
pET-PgpA C75V	This study	
pET-PgpA C69V	This study	
pET-PgpA D86A	This study	
pET-PgpA D93A	This study	
pET-PgpA E94A	This study	
pET-PgpA R121A	This study	
pKD3	(44)	
pKD46	(44)	
pSIM6	(45)	
pDSW195	Gift from William Margolin	
pTrc-PgpA	This study	Generated using pDSW195

Table S2. Oligos and Sequences for Genetic Mutant Strains

**Oligos**	
pgpA KO forward	TCACATCAGGCATCGGTGCACAACTACGACAGAATACCCAGCGGCCAGTGatgggaattagccatggtcc
pgpA KO reverse	CCGCCGATATCGAAGGGCTTTGTTTTATTCGTGACGGCGAACCTGTTACAtgtaggctggagctgcttcg
pgpA 50bp+KN1 Forward	atgaccattttgccacgccataaagatgtcgcgaaaagtcgcctgaagaATAGGAACTTCAAGATCC
pgpA 50bp+ccdB1 reverse	ccagtgatgaccgataaaatacaggatgcctgcggaaatcaccccggcgTTATATTCCCCAGAACATCAGG
pgpA Forward	atgaccattttgccacgc
pgpA Reverse	ccagtgatgaccg
**Sequencing result**	
W3110 *ΔpgpA*	NNNNNNNAAACNCNNCCNNNTNNNNNNNNANNNGNNNCNNNNNNNNNNGNNNNGTNNNNTNNGATNGNNNNANTNGNNANANNNNNNNGCCNNGANGNTNNCATNNCCANNNANNNCNNCTTAGNCCTNTNNGANNNNNNTNCTNTNNNNNANNGNNNTGAANNNNTCCNGCTACANNAACAGTTNGCCNTCNNGANTAAAACAAAGCCNNGNNNTNGNGTCATTTNNCGATACAGTAAACGTANGCCCAGGNNNCGAGNGCCACATCCAGCGCGCACGTTCAGTTCCGCACAGNGAAACACNANTCGTNATCCTCACNCCAGAGAGCGCCNNNGCAGCNCTNNTNNNNTTCAACANGCGAGANAGNNNTCAGAAAANNACAGAANNGGAANNTCANNNAATGCGCNCNGGNGNNGNNGNNTTNNNNNNNNNNNNNNNNNNNTTTTTNTGNNNNNNNGTGAAAGCCAGCGACTGCGGCGCACGTATTGACCTGGCATTGCTGCCGTTTTCTGATGCGCTTTCTCGCCATGTTGAACCGGAACAGGCGCTGCGCTGGGCGCTCTCTGGCGGTGAAGATTACGAGTTGTGTTTCACTGTGCCGGAACTGAACCGTGGCGCGCTGGATGTGGCTCTCGGACACCTGGGCGTACCGTTTACCTGTATCGGGCAAATGACCGCCGATATCGAAGGGCTTTGTTTTATTCGTGACGGCGAACCTGTTACATGTAGGCTGGAGCTGCTTCGAAGTTCCTATACTTTCTAGAGAATAGGAACTTCGGAATAGGAACTAAGGAGGATATTCATATGGACCATGGCTAATTCCCATCACTGGCCGCTGGGTATTCTGTCGTAGTTGTGCACCGATGCCTGATGTGACGCTTGTCACGTCTCATCAGGCCTGGACTCTTATTTAAATCCTACGACAGGATGCGGTTTATACGGCGTTTCCAGTTCGGCAATCTGTTCCGGCTTCAAAGTGATATCCACCGCGTTCAATAGCTCATCAAGCTGTTCTTCGCGCGAAGTTCCGATAATCGGTGCGGCAATGCCCGGTTTACTCAACAACCAGGCCAGCGCAACTTGTGCTCGTGTCGCCCCCAGTCTCACNGANGCNNNNNNNNNNN

pgpA-C75V strain	NNNNNNNNNNTCNCCCGGCGANATATCGTCGATCATGATCCCCATG<br>CCGCCATGCACATTGCGATCAAACCAGCGGATCGGCCACGGCTTCC<br>ACATATCCAGAATACGGAAAATCACAAACCCGGCGGCAACCCACTG<br>CCAGTCATTGGTCGGCAGCGCCATGAGCGTGATCCACATACCAATA<br>AATTCGTCCCAGACAATGCTGCCATGATCGTGCACACCATGTCTTTC<br>GCCGTTTGATGGACAAGATAGACGCCGATACAGATCCCCAGCATCA<br>CCACCAGCGAGTAGAGCTGCCAGGGCAAAAAGGTCATCAGATACCA<br>GAACGGAATCGCTGCCAGCGAGCCCATCGTCCCAGGAACGATCGGG<br>CTTAATCCACTTCCGAATCCGACAGCAAGTAGATGCCACGGATTACT<br>CATCTTCAGGCGACTTTTCGCGACATCTTTATGGCGNGNNNAANGGG<br>GTCANNNNNNAGAAGGNCNNNAAAAGTCCCTGAAGATGANTAATC<br>CATGGCATCTACTTGCTGTCGGATTCNGAAGTGGATTANNCCCGATC<br>GTTCCTGGNACNATNGGCTCNCTGGCANCGATTCCNTTCTGGTATCT<br>GATGACCTTTTTGCCCTGGNAGCTCTACTCNCTNGTGGNNATGCTGG<br>GGNTCTGTATCNGNNNCTATCTNNTCNTCAAACGGCCAAAGNNTGG<br>GNNNGCACNATCATNGNANCATNNNCTGGNACGAANTTATNGGNTA<br>TGATGGNTCNNNCCNGGGCCNGCNACNNGNNCGGNNNNGNNNNNC<br>NCNGNNNNNNNANNNCNNNNNNNNNANTNNNNANCCNTGNCNAT<br>CNNNNNNNTNNATCNNNNNNNNNNNNGGCNNNCNNNGNGGNANNN<br>NNNNNANNNNNNNNANNNTNTNNNNN

Data S1. (separate file)

Sequence alignment of 316 PgpA homologs showing the conservation of the residue Cys75 (highlighted using a dashed red square) in β- and γ-proteobacteria.

Data S2. (separate file)

Metabolic analysis of *E. coli* W3110 wild type, Δ*pgpA*, and *pgpA-C75V*. Bacterial cultures were cultivated using 1% glucose. Intracellular metabolites were extracted from the same number of cells. Metabolite levels were measured in three biological replicates per condition; fold changes were calculated using the median, and *p*-values were determined using unpaired two-tailed Student's t-tests on individual replicate values.
