# Supplementary material for: A redox-sensitive phosphatase regulates glycolysis as a metabolic switch in the bacterial inner membrane
Source: Sci Adv. 2026 May 6;12(19):eaea8724. doi: 10.1126/sciadv.aea8724 (PMC13148334; doi:10.1126/sciadv.aea8724)
Supplement: Supplementary file 2 — Data S1 and S2 [file sciadv.aea8724_data_s1.pdf › sciadv.aea8724_data_s1.pdf]

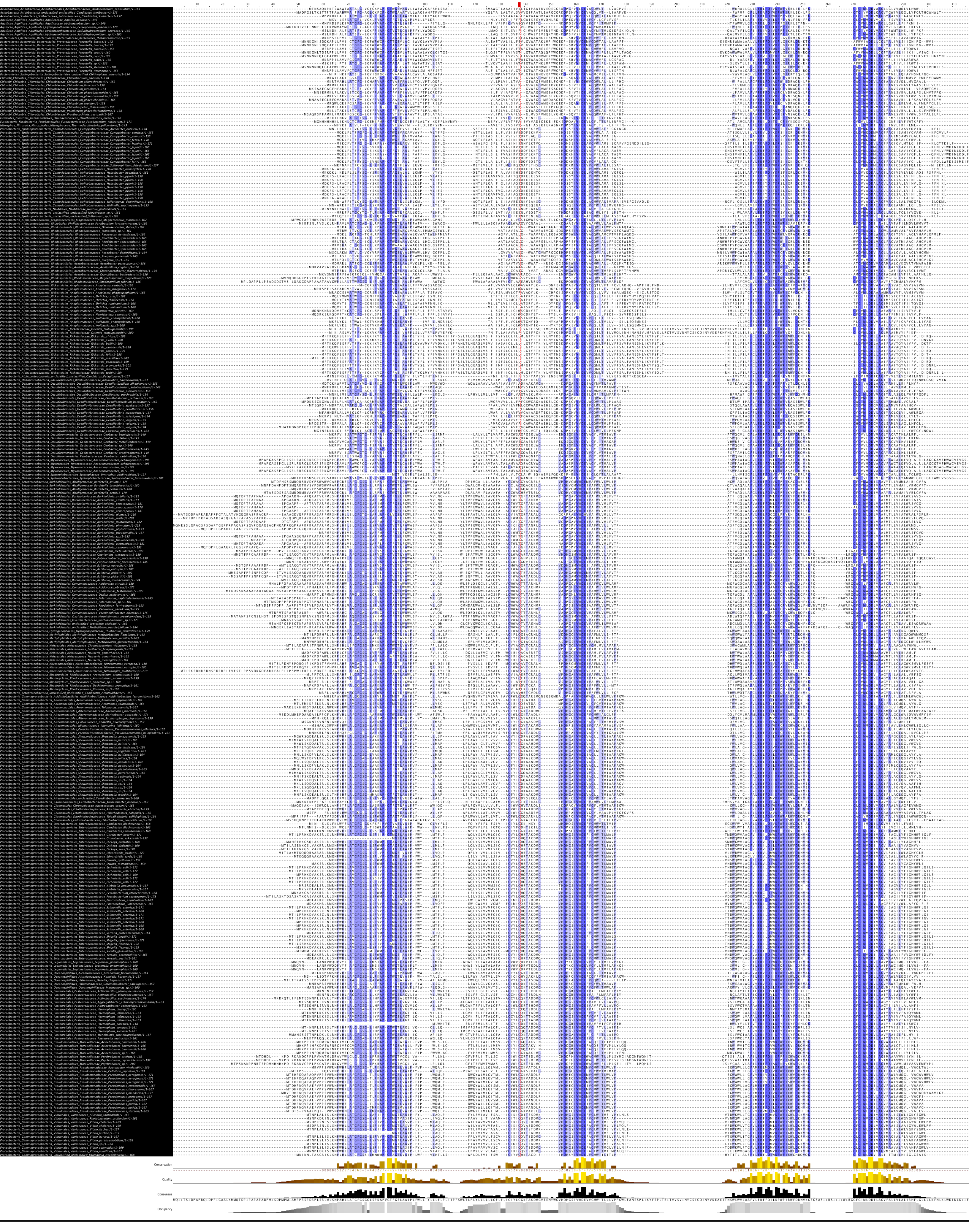
Conservation
Quality
Consensus
Occupancy
